# Valorization of *Caryocar brasiliense* Byproducts: Microwave-Assisted Extraction of Phenolics and Material Characterization for Environmental and Bioenergy Applications

**DOI:** 10.1021/acsomega.5c09005

**Published:** 2025-11-20

**Authors:** Mariele Dalmolin da Silva, Eliza Araujo Martins, Renata Pereira Lopes Moreira, André Pereira Rosa, Alisson Carraro Borges

**Affiliations:** † Department of Agricultural Engineering, Federal University of Viçosa, 36570-900 Viçosa, Minas Gerais, Brazil; ‡ Department of Chemistry, Federal University of Viçosa, 36570-900 Viçosa, Minas Gerais, Brazil

## Abstract

The exploitation of the pequi (*Caryocar brasiliense*) fruit generates underutilized agro-industrial residues, such as endocarps and almonds, which are rich in value-added compounds. In addition to these residues, other parts of the plant, such as the bark, also present technological potential that remains largely unexplored. This work aimed to physicochemically characterize these byproducts to evaluate their potential for environmental and industrial applications within the context of the bioeconomy and circular economy. Almonds (AM), thorny endocarp (PTE), a mixture of endocarp and almond (PTEA), and pequi tree bark (PTB) were analyzed using physicochemical, thermal, textural, and morphological characterization techniques. The results revealed significant contents of lignin (up to 34.26%), holocellulose (up to 56.25%), and lipids (up to 47.82%), as well as the presence of phenolic compounds (193.73 mg_GAE_ g^–1^), with potential for use as natural coagulants. The materials exhibited compact morphology and predominantly mesoporous structures (2–50 nm), with specific surface areas ranging from 0.32 to 0.71 m^2^ g^–1^. Analysis of the solid residues after microwave-assisted extraction indicated structural changes that facilitate the release of bioactive compounds. PTB stood out for its lignocellulosic composition and high content of tannins and phenolic compounds, while AM showed a high lipid content, demonstrating viability for bio-oil production. These findings highlight the potential of these materials for clean and sustainable technologies, contributing to the mitigation of environmental impact and generating value in regional production chains.

## Graphical abstract

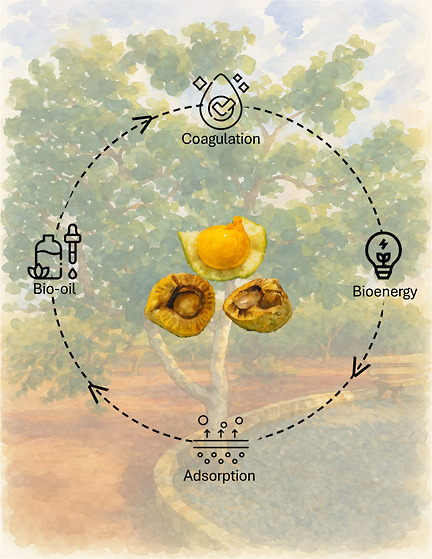

## 1 Introduction

The Cerrado is the second-largest biome in Brazil and the most extensive neotropical savanna in South America. Renowned for its remarkable biodiversity, this biome covers around 23% of the Brazilian territory, making it one of the country’s most important ecological regions.^1^ The Cerrado vegetation comprises species highly adapted to harsh environmental conditions, such as elevated temperatures, prolonged drought, nutrient-poor soils, intense ultraviolet radiation, and frequent natural fires.^2^

Among the native species thriving in this challenging environment, *Caryocar brasiliense*, popularly known as pequi, stands out for its exceptional ecological, cultural, and economic relevance. The bark of the pequi tree is highly valued in the Brazilian Midwest, where it is used as a raw material in diverse production chains, ranging from small-scale extractive communities to medium-sized industries.^3^ The fruit is composed of approximately 75–84% peel, 14–16% thorny endocarp, 4–7% pulp, and 0.5–1% kernel, with these proportions varying depending on the region.^4^

Pequi plays a key role in both local and national economies. In 2023, extractive production of the fruit reached approximately 51,000 tons, with the states of Minas Gerais, Goias, and Tocantins ranking among the leading producers in Brazil.^5^ The market for pequi-derived products has expanded significantly, with a 127.9% increase in production volume and a 122.7% rise in commercial value between 2019 and 2021, driven by the valorization of its byproducts and broader access to new consumer markets.^6^ The diversification of commercial formats, such as frozen pulp, preserves, nuts, oil, and flour, has further strengthened pequi’s presence in both regional and national markets.^7^

Despite its economic importance, pequi processing generates a large volume of agro-industrial waste. The fruit peel accounts for approximately 80% of its mass and, like the thorny endocarp, is often discarded, contributing to environmental impacts such as leachate generation, greenhouse gas emissions, and soil contamination.^7,8^ The high proportion of lignocellulosic components in this waste hinders its reuse, leading to the loss of bioactive compounds with industrial potential.^9^

Although commonly discarded, pequiresidues hold significant potential for the recovery of valuable bioactive compounds, such as phenolics and tannins.^10^ These compounds have demonstrated effectiveness as natural coagulants in the removal of various pollutants, including chemical oxygen demand (COD), turbidity, color, suspended solids, total phosphorus, algae, and heavy metals.^11,12^ Plant species such as *Acacia mearnsii*, Quebracho, and *Castanea sativa* are widely used as commercial sources of these compounds.^13,14^ In Brazil, the Tanfloc product line, developed by TANAC, exemplifies the industrial use of condensed tannins, extracted from *A. mearnsii* bark, for efficient wastewater treatment applications. Tomasi et al.^12^ highlight the effectiveness, biodegradability, and lower environmental impact of tannin-based coagulants compared to conventional chemical agents. Despite their potential, studies focusing on the extraction and characterization of these compounds from native Cerrado residues, such as pequi byproducts, remain scarce. This gap is particularly evident in the context of circular economy strategies, underscoring the innovative character and relevance of exploring these underutilized biomasses.

Recent studies have highlighted the promising energy potential of pequi residues, especially the thorny endocarp. Miranda et al.^15^ indicated its suitability for biochar with properties favorable for energy applications. Ghesti et al.^8^ proposed an integrated approach combining pyrolysis, gasification, and transesterification to obtain biochar, syngas, and bio-oil, where prior oil extraction from kernels improved product quality and illustrated the valorization potential of these lignocellulosic residues within a circular economy framework. Public policies and environmental regulations play a key role in enabling the sustainable use of pequi tree bark. The enactment of Law 15,089 in January 2025 established guidelines for responsible pequi exploitation, including reforestation, product certification, and support for extractive communities. These initiatives aim to preserve the species while encouraging sustainable value chains and enhancing economic autonomy for small producers.^16^

Considering these aspects, this work aims to comprehensively characterize the biomass of pequi tree byproducts (*C. brasiliense*) both before and after microwave-assisted extraction of phenolic compounds, with a detailed discussion of their physicochemical properties. The focus is on evaluating their potential for diverse applications, particularly emphasizing the extraction of these bioactive compounds, while also assessing their suitability as natural coagulants, adsorbents, and in thermochemical processes within a circular economy framework.

## 2 Materials and Methods

### 2.1 Standards and Reagents

The following chemicals were used in this study: sodium chloride (NaCl, ≥99.0% purity, ACS, Brazil), hydrochloric acid (HCl, 37% purity, PA ACS, Fmaia, Brazil), sodium hydroxide (NaOH, micropellet, ≥97% purity, PA, Synth, Brazil), sodium carbonate (7.5% w/v, ≥99.5% purity, Dinâmica, Brazil), analytical standard of gallic acid (≥98% purity, Sigma-Aldrich, Steinheim, Germany), and Folin–Ciocalteu’s phenol reagent (1.9–2.1 N, ≥98% purity, Sigma-Aldrich, Steinheim, Germany). Absolute ethanol (≥99.5% purity, Synth, Brazil) and deionized water were used as solvents.

### 2.2 Obtaining the Byproducts of *C. brasiliense*

Four byproducts of *C. brasiliense* (Figure 1) were selected for investigation: almond (AM, i.e., the pequi fruit kernel), thorny endocarp (PTE), thorny endocarp with almond (PTEA), and pequi tree bark (PTB). The bark and fruits were harvested directly from *C. brasiliense* trees located in the northern region of Minas Gerais, Brazil. After collection, samples were stored in plastic bags and frozen at −18 °C until further processing. The fruit pulp was manually removed, and the thorny endocarp was carefully separated from the almonds using a knife. Subsequently, all materials were oven-dried at 65 °C for 72 h in a forced-air circulation oven (Marconi, MA035, Brazil), ground using a Willey-type knife mill, and sieved to obtain particle sizes between 40 and 80 mesh. The almonds underwent additional grinding in a household blender (Fama, Brazil) for 2 min to ensure finer particle size. The processed byproducts were then prepared for subsequent physicochemical characterization and bioactive compound extraction.

**Figure 1:**
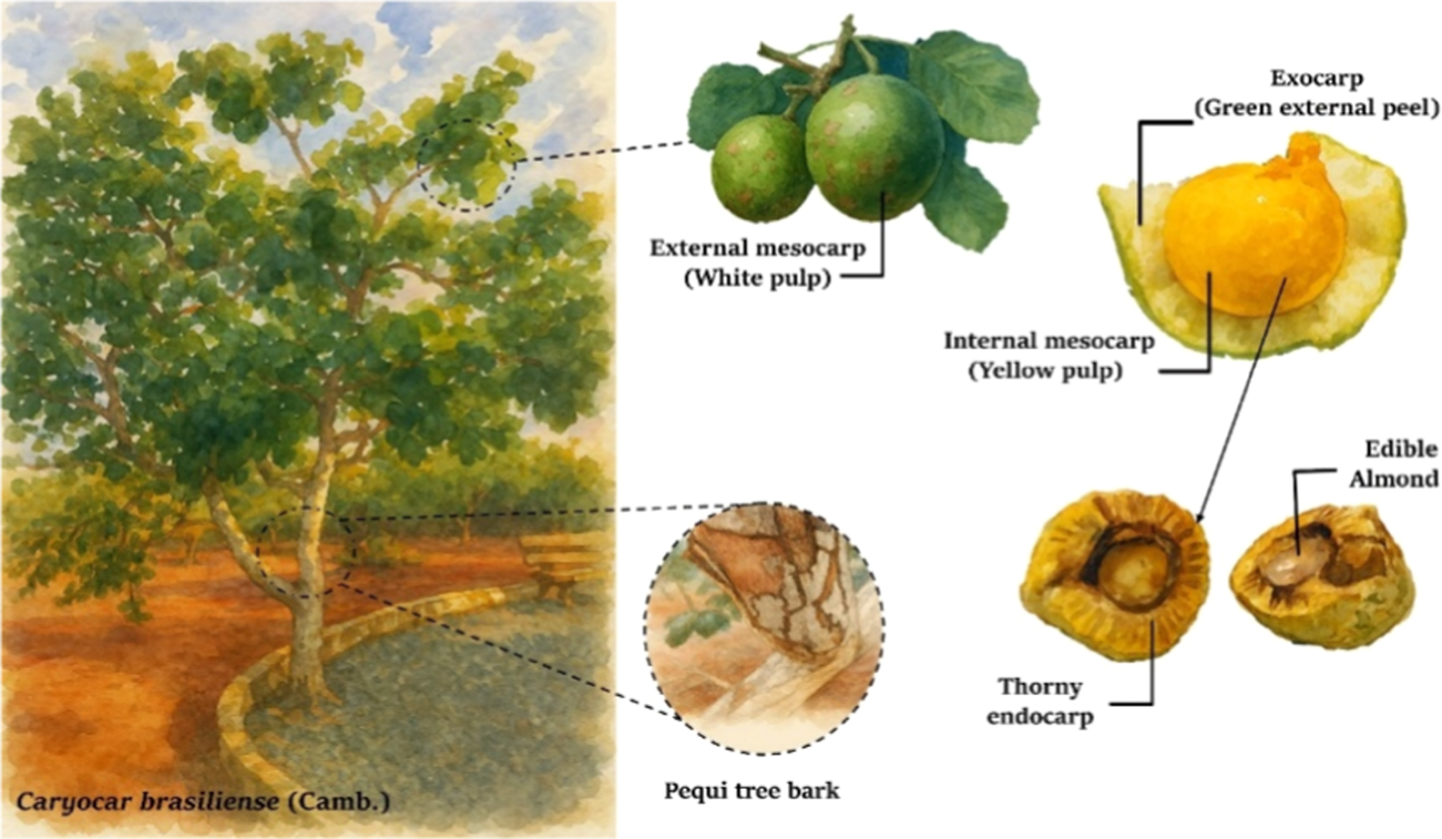
General view of the pequi (*Caryocar brasiliense*).

### 2.3 Characterization of *C. brasiliense* Byproducts

Structural chemical analysis was conducted to quantify holocellulose and lignin, both acid-insoluble and acid-soluble lignin and extractives contents, in the studied materials. Acid-insoluble and acid-soluble lignin were determined following the standardized TAPPI T222 om-97 and UM 250 standard methods, respectively. Holocellulose content was calculated by difference. Preparation of extractive-free pequi byproduct samples was performed according to the TAPPI T264 cm-97 standard method.^31^

Carbohydrate fractions, including arabinans, galactans, glucans, xylans and mannans, were quantified according to the SCAN-CM 71:09 method, using an ion chromatograph (940 Professional IC Vario, Metrohm, Switzerland).

Proximate analysis was conducted according to ASTM D1762-84, with some modifications. A porcelain crucible was used instead of platinum, and volatile matter analysis was conducted at 950 °C. The sample and crucible were initially placed on the muffle furnace lid for 2 min, followed by 7 min inside the furnace, totaling 9 min of exposure. Oxygen content was calculated by difference. Protein content was determined via the Kjeldahl method following Tedesco et al.^17^ using a nitrogen-to-protein conversion factor of 5.18 for almonds (pequi kernels) and 6.25 for the other samples. Total lipid content was measured by Soxhlet (Marconi, MA-188) extraction according to.^18^

Elemental analysis of carbon, hydrogen, nitrogen, and sulfur (CHNS) was performed using an elemental analyzer (LECO, TruSpec Micro, USA). Oxygen content was again determined by difference. The higher heating value (HHV) was measured using an adiabatic bomb calorimeter (Parr, USA) in accordance with NBR 8633.^19^

For X-ray diffraction (XRD) analysis, measurements were conducted using a diffractometer (Bruker D8-Discover, Germany) equipped with a copper tube and Goebel mirror, using Ni-filtered Cu Kα radiation (λ = 1.5418 Å). Scanning was performed at a rate of 0.05° s^–1^ over a 2θ range of 5° to 60°.

Scanning electron microscopy (SEM) was used to assess the surface morphology of the materials. Images were captured using a scanning electron microscope (JEOL, JSM-6010LA, Japan).

Zeta potential (ZP) analysis was performed using a particle analyzer (Anton Paar, Litesizer 500, Austria). For the measurements, 0.25 g of each residue was dispersed in 250 mL of ultrapure water and sonicated in an ultrasonic bath (Eco-Sonics, Ultronique, Brazil) for 120 min. Then, 10 mL of this solution was collected, and the pH was adjusted to values between 2 and 12 (2, 4, 6, 8, 10, and 12) using 0.1 mol L^–1^ HCl or NaOH. The samples were further sonicated for 3 min and transferred to a zeta potential cuvette for analysis. All assays were performed in duplicate.

The point of zero charge (pH_PZC_) was determined as described by Akkari et al.^20^ For this, 0.15 g of sample was added to 50 mL of NaCl solution (0.01 mol L^–1^) in each flask, with pH adjusted between 2 and 12 using 0.1 mol L^–1^ HCl or NaOH. The solutions were stirred at 200 rpm for 24 h at 25 °C on an orbital shaker. At the end of the contact time, the final pH of the samples was measured using a pH meter. The pH_PZC_ was identified as the intersection point of the final pH versus the initial pH curve with the *x*-axis, where ΔpH equals zero. This procedure was performed in duplicate.

Thermogravimetric analysis (TGA) and differential thermogravimetry (DTG) were employed to assess the mass loss of biomass components relative to temperature. Samples, sieved to a 100-mesh particle size and weighing approximately 2 mg, were analyzed using a thermobalance under an inert nitrogen flow (50 mL min^–1^). Thermograms were recorded from 30 to 800 °C at a controlled heating rate of 10 °C min^–1^.

The specific surface area and pore characteristics of the pequi materials were analyzed by N_2_ gas adsorption isotherms at 77 K using a surface area and porosity analyzer (Anton Paar, Nova 600, USA). The specific surface area was determined using the Brunauer–Emmett–Teller (BET) method, while the Barrett, Joyner, and Halenda (BJH) method was employed for the pore size distribution. Prior to analysis, the materials were degassed under vacuum at 100 °C for 3 h to remove impurities.

### 2.4 Total Phenolic Content (TPC)

Extracts from the AM, PTE, PTEA, and PTB materials were obtained using 5 mL of 50% (v/v) ethanol. The extraction was performed in a single-mode microwave synthesis reactor (CEM Matthews-NC, Discover System, USA) operating at a frequency of 2.455 MHz. During the experiments, the temperature was ramped over 4 min to reach the target of 105 °C, starting with an initial power of 80 W. The extraction conditions: the solid-to-liquid ratio of 30 mL g^–1^, extraction time of 4 min, and temperature of 105 °C, were selected based on previous studies reported in the literature^21,22^ to conduct a preliminary assessment of the materials’ extractive potential.

The total phenolic compounds (TPC) were quantified using the Folin–Ciocalteu colorimetric assay, following the methodology proposed by Singleton et al.^23^ with adaptations and Silva et al.^21^ In this procedure, 200 μL of the previously diluted extract (1:5 dilution for PTE and PTEA, and 1:10 for PTB) were mixed with 1000 μL of the Folin–Ciocalteu reagent (diluted 1:10). After an initial 5 min resting period, 800 μL of 7.5% (w/v) sodium carbonate solution were added, and the mixture was then incubated at 50 °C for 30 min in the dark.

Absorbance was measured at 765 nm for TPC using a UV–vis spectrophotometer equipped with a 96-well quartz microplate. Measurements were performed in triplicate using a microplate reader (Multiskan GO, Thermo Scientific, Germany). TPC quantification were based on analytical curves constructed with gallic acid standards, respectively, in the concentration range of 0 to 100 mg mL^–1^. Results were expressed as milligrams of gallic acid equivalent per gram of dry sample (mg_GAE_ g^–1^) for TPC.

All experimental results are expressed as means of duplicates (*n* = 2) and reported as mean ± range, where the “range” reflects only technical variability.

### 2.5 Structural and Morphological Characterization

The functional groups present in the extracts of pequi byproducts were analyzed by Fourier transform infrared spectroscopy (FTIR) using the attenuated total reflectance (ATR) technique. The equipment used was a PerkinElmer (Frontier Single Range-MIR, USA) in transmittance mode, scanning in the region from 550 to 4000 cm^–1^. The FTIR spectra of the raw biomass samples prior to microwave-assisted extraction have been previously reported in Silva et al.^21^

The surface morphology of the samples after extraction was examined using scanning electron microscopy (SEM). Micrographs were obtained using a (JEOL, JSM-6010LA, Japan). These analyses provided qualitative information on surface texture, particle aggregation, and structural changes induced by microwave-assisted extraction.

## 3 Results and Discussion

### 3.1 Chemical Analysis of *C. brasiliense* Byproducts

Table 1 shows the structural chemical composition, including carbohydrates, proteins, and lipids, of pequi byproducts (AM, PTE, PTEA, and PTB).

**Table 1:** Structural Chemistry and Carbohydrate Analysis of Pequi Almonds (AM), Pequi Thorny Endocarp (PTE), Pequi Thorny Endocarp + Almonds (PTEA) and Pequi Tree Bark (PTB)

	AM	PTE	PTEA	PTB
Structural Chemical Analysis (wt % Dry Basis)^b^				
holocellulose	12.55 ± 0.14	35.72 ± 0.06	38.22 ± 0.71	56.25 ± 0.10
insoluble lignin	8.40 ± 0.62	27.29 ± 0.73	25.51 ± 0.33	34.26 ± 0.03
soluble lignin	6.03 ± 0.19	1.68 ± 0.07	1.83 ± 0.11	1.50 ± 0.03
total lignin	14.43 ± 0.44	28.97 ± 0.66	27.34 ± 0.44	35.77 ± 0.06
extractives	73.03 ± 0.16	35.31 ± 0.79	34.44 ± 1.86	7.98 ± 0.25
total carbohydrate^a^ (%)	2.06 ± 0.06	27.98 ± 0.34	27.67 ± 0.72	43.62 ± 0.16
arabinans	0.40 ± 0.03	0.13 ± 0.13	0.33 ± 0.01	1.66 ± 0.01
galactans	0.13 ± 0.01	1.00 ± 0.02	1.21 ± 0.01	0.97 ± 0.05
glycans	1.21 ± 0.06	20.51 ± 0.32	20.95 ± 0.69	33.27 ± 0.14
xylans	0.31 ± 0.02	6.34 ± 0.01^b^	5.18 ± 0.21	7.09 ± 0.07
mannans	-	-	-	0.64 ± 0.01

a By difference.

b Mean ± range.

Structural analysis identified holocellulose, soluble and insoluble lignin, total lignin, and extractives in varying proportions, influenced by factors such as species, age, and development stage.^24^ PTB exhibited the highest holocellulose content (56.25%), followed by PTEA (38.22%), PTE (35.72%), and AM (12.55%). Holocellulose, composed of cellulose and hemicellulose, is important for biochemical conversion, enhancing enzymatic digestion and production of reactive bioproducts.^25^ For thermochemical applications (pyrolysis, gasification), biomass with high lignin and low holocellulose favors bio-oil production. Thus, AM shows greater potential, while PTB has the highest total lignin content. Lignin consists of aromatic alcohol units (*p*-coumaryl, coniferyl, sinapyl) and functional groups (methoxy, carbonyl, hydroxyl, aromatic rings) that confer chemical reactivity and adsorption capacity.^26^ Its aromatic nature also promotes biochar formation with high fixed carbon and surface area, essential for adsorption, and enables interactions with pollutants via hydrogen bonding, hydrophobic forces, π–π stacking, and electrostatic attractions.^27^

AM had the highest extractive content (73.03%) and PTB the lowest (7.98%). Extractives aid lignin degradation, forming phenolic and volatile compounds. High extractive levels improve energy value and bio-oil yield in pyrolysis, whereas low levels enhance biochar thermal stability.^28^ Considering its extractives and H/C ratio (Table 2), AM is a promising bio-oil feedstock.

**Table 2:** Chemical Characteristics of Pequi Almonds (AM), Pequi Thorny Endocarp (PTE), Pequi Thorny Endocarp + Almonds (PTEA), and Pequi Tree Bark (PTB)

	AM	PTE	PTEA	PTB
Proximate Analysis (wt % Dry Basis)^c^				
volatile matter^a^	90.22 ± 0.23	81.57 ± 0.26	83.82 ± 0.01	77.98 ± 0.04
ash^a^	5.06 ± 0.10	0.86 ± 0.01	1.81 ± 0.01	1.50 ± 0.01
fixed carbon^a^ ^,^ ^b^	4.72 ± 0.13	17.57 ± 0.25	14.37 ± 0.01	20.51 ± 0.05
moisture	8.61 ± 0.21	15.59 ± 0.04	14.13 ± 0.10	21.86 ± 0.06
proteins (%)	22.11 ± 0.01	7.19 ± 0.01	9.70 ± 0.01	3.29 ± 0.01
lipids (%)	47.82 ± 0.32	27.50 ± 0.77	31.49 ± 1.03	2.03 ± 0.48
Ultimate Analysis (wt % Dry Basis)				
C	56.09 ± 0.20	52.19 ± 0.01	52.11 ± 0.03	45.42 ± 0.01
H	8.39 ± 0.17	6.80 ± 0.03	7.15 ± 0.05	4.69 ± 0.09
N	4.25 ± 0.01	1.36 ± 0.04	1.29 ± 0.02	0.51 ± 0.04
S	2.07 ± 0.01	1.64 ± 0.01	1.91 ± 0.01	2.01 ± 0.02
O^b^ ^b^	29.1 ± 0.02	38.02 ± 0.06	37.54 ± 0.03	47.37 ± 0.01
H/C	1.78 ± 0.18	1.55 ± 0.02	1.64 ± 0.04	1.23 ± 0.05
O/C	0.39 ± 0.02	0.55 ± 0.04	0.54 ± 0.03	0.78 ± 0.01
Higher Heating Value (HHV) (MJ kg^–1^)				
	27.54 ± 0.32	23.16 ± 0.17	24.15 ± 0.18	22.15 ± 0.02

a Dry base.

b By difference.

c Mean ± range.

Carbohydrate content ranged from 2% to 43% (Table 1). Biomass with ≥25% carbohydrates is suitable for bioproduct synthesis.^29^ PTB showed the highest glucan (33.27%), followed by PTEA, PTE, and AM, indicating its biotechnological potential. Xylan was the second most abundant polysaccharide, especially in PTB (7.09%). Minor polysaccharides (galactans, arabinans, mannans) were below 2%. Xylan, a branched noncellulosic polysaccharide with many hydroxyl groups, can be chemically modified to enhance metal ion adsorption, making it a promising adsorbent.^30^

### 3.2 Proximate, Lipids, and Proteins, Elemental, and Calorific Value Analysis

Table 2 presents the proximate, lipids, and proteins, elemental, and calorific analyses of the *C. brasiliense* byproducts. All samples showed high volatile matter content (>77%), indicating a significant presence of volatile organic compounds such as H_2_, CO, CO_2_, CH_4_, N_2_, and hydrocarbons.

According to Nascimento-Silva et al.^4^ volatile contents above 74% are considered high, resulting in increased reactivity and volatility, which favor the production of noncondensable gases such as bio-oil through pyrolysis. The *C. brasiliense* byproducts exhibited fixed carbon contents ranging from 4.72% to 20.51% and ash contents between 0.86% and 5.06%. These parameters are critical for adsorption performance. High fixed carbon enhances contaminant retention capacity, while low ash content reduces the presence of inorganic oxides that could impair adsorption efficiency.^32^

Ash contents above 7% reduce the suitability of raw materials for energy applications such as direct combustion, pellet production, and briquetting, as they can lower boiler efficiency due to deposit buildup on heat exchange surfaces.^33^ For comparison, ash levels between 7% and 10% have been reported in woody biomass like black wattle bark, red oak, and willow (*Salix* spp.), which limits their viability for energy use.^34^

Moisture content in the analyzed materials ranged from 8.61% to 21.86%. High moisture levels can be detrimental to thermochemical processes, as contents above 10% reduce pyrolysis efficiency by lowering bio-oil yield and quality, delaying ignition, and decreasing calorific value due to the additional energy required for water evaporation.^33^ However, in microwave-assisted processes, higher moisture can be advantageous, as water efficiently absorbs microwave energy and enhances gasification reactions, favoring syngas production.^35^

Protein and lipid contents of the pequi byproducts are shown in Table 2. AM exhibited the highest lipid content (47.82%), indicating strong potential as a source of vegetable oil. For comparison, soybeans, one of the most widely cultivated oilseeds, contain 8.1% to 24% lipids on a dry weight basis.^36^ According to De Lima et al.,^37^ pequi grows in regions with intense solar radiation, which promotes the formation of free radicals and stimulates the biosynthesis of antioxidant compounds such as phenolics and carotenoids. Similar trends have been reported in the literature. Machado et al.^38^ found lipid and protein contents of 30.02% and 17.52% in almonds, and 19.13% and 2.93% in thorny endocarp, respectively. The values observed in the present study surpass those previously reported, likely due to geospatial variation, which influences fruit composition, including moisture, lipid content, energy density, and yield. Among the samples, only PTB showed low lipid content (2.03%), consistent with its protective role in the tree, unlike fruits and almonds that serve primarily as energy reserves.^39^

Elemental analysis (Table 2) showed carbon and oxygen as the major components (45.42–56.09% and 29.19–47.37%, respectively), consistent with values found in forest and agricultural residues.^15^ High carbon content favors thermochemical conversion by increasing heat release and improving gasification efficiency. Hydrogen was higher in AM and PTEA (4.69–8.39%), while nitrogen and sulfur levels were low (0.51–4.58% and 1.64–2.07%), which is environmentally advantageous, as it reduces emissions of toxic gases (H_2_S, SO_*x*_, NO_*x*_) during pyrolysis.^40^ Overall, the materials exhibit good potential for thermochemical applications.

The H/C and O/C ratios from ultimate analysis (Table 2) are key indicators of saturation, aromaticity, and oxygenation in biomass, allowing the prediction of thermochemical behavior.^41^ High H/C and low O/C denote higher reactivity and energy density, while the opposite reflects greater aromaticity, oxygenation, and lower energy yield. AM showed the highest H/C (1.78) and lowest O/C (0.39), consistent with its elevated C and H contents, lower O content, and superior HHV (27.54 MJ kg^–1^), likely related to its higher lipid content.

The HHV of the *C. brasiliense* byproducts ranged from 22.15 to 27.54 MJ kg^–1^; moisture reduced energy yield by increasing the energy required for evaporation, and excess oxygen, although supporting combustion, lowered net energy release. Despite the absence of thermal pretreatment, these values are comparable to those of charcoal (25–35 MJ kg^–1^), underscoring the high bioenergy potential of these materials.^42^

### 3.3 XRD and SEM Analyses of the Byproducts

Figure 2 shows the XRD diffractograms of pequi byproducts (AM, PTE, PTEA, and PTB), revealing typical lignocellulosic carbonaceous structures. All samples exhibited a broad peak between 20° and 30°, indicative of amorphous carbon composed of aromatic carbon sheets. The 002 plane peak appeared near 2θ = 19.82° (AM), 21.38° (PTEA), 20.56° (PTE), and 14.88° (PTB), with PTEA and PTB showing a slight shift to higher angles (2θ = 22.14°), suggesting increased amorphous character. Since cellulose is crystalline and hemicellulose and lignin are amorphous, this amorphous nature favors pollutant adsorption due to higher surface area and abundant active sites.^43^

**Figure 2:**
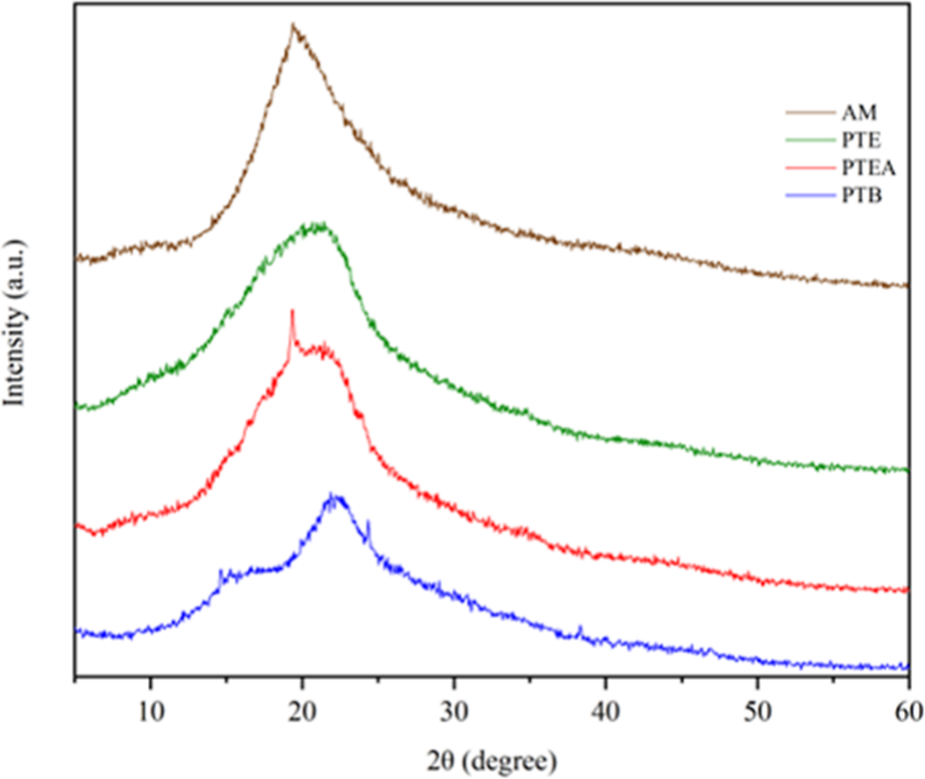
XRD diffractograms of pequi almonds (AM), pequi thorny endocarp (PTE), pequi thorny endocarp + almonds (PTEA) and pequi tree bark (PTB).

SEM analysis was conducted to further evaluate surface morphology (Figure 3). The images reveal that PTE and PTEA exhibit similar agglomerate and uniform surfaces, with PTEA appearing more compact and globular, likely due to the presence of almonds. In contrast, PTB shows a rough, coarse, and dense texture, while AM presents a comparatively smooth and homogeneous surface. According to Chaouki et al.,^44^ materials with lamellar, agglomerated, and irregular porous surfaces favor adsorption of organic molecules. Similarly, Kumari et al.^45^ highlighted that fine, overlapping, and wrinkled surfaces enhance contaminant adsorption. These morphological features also improve coagulation by facilitating contaminant capture. Conversely, AM’s smoother surface may limit its adsorption and coagulation efficiency due to fewer active sites.

**Figure 3:**
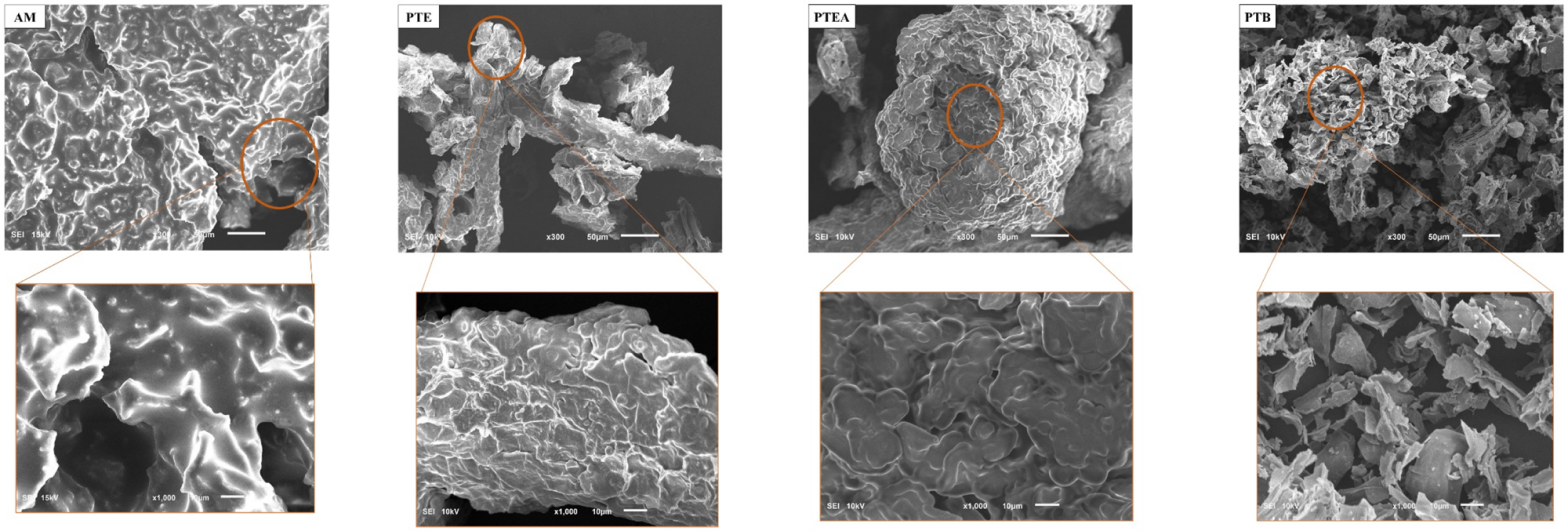
SEM images of pequi almonds (AM), pequi thorny endocarp (PTE), pequi thorny endocarp + almonds (PTEA), and pequi tree bark (PTB), obtained at 300× (top) and 1000× (bottom) magnifications.

### 3.4 Zeta Potential (ZP) and Point of Zero Charge (pH_PZC_) Analyses

Figure 4a shows the zeta potential (ZP) values of AM. PTE, PTEA, and PTB as a function of pH. ZP arises from the protonation and deprotonation of polar functional groups like hydroxyl (OH), carboxyl (COO^–^), and amino groups on the material surface. All materials exhibited negative ZP values at neutral pH: AM (−32.10 ± 1.27 mV), PTE (−37.33 ± 0.78 mV at pH 5.23), PTEA (−36.50 ± 0.53 mV at pH 5.38), and PTB (−25.08 ± 2.62 mV at pH 4.89). At higher pH, increased dissociation of OH and COO^–^ groups lead to more negative surface charges, while at pH below 4, protonation suppresses dissociation and ZP becomes positive for PTE and PTEA.

**Figure 4:**
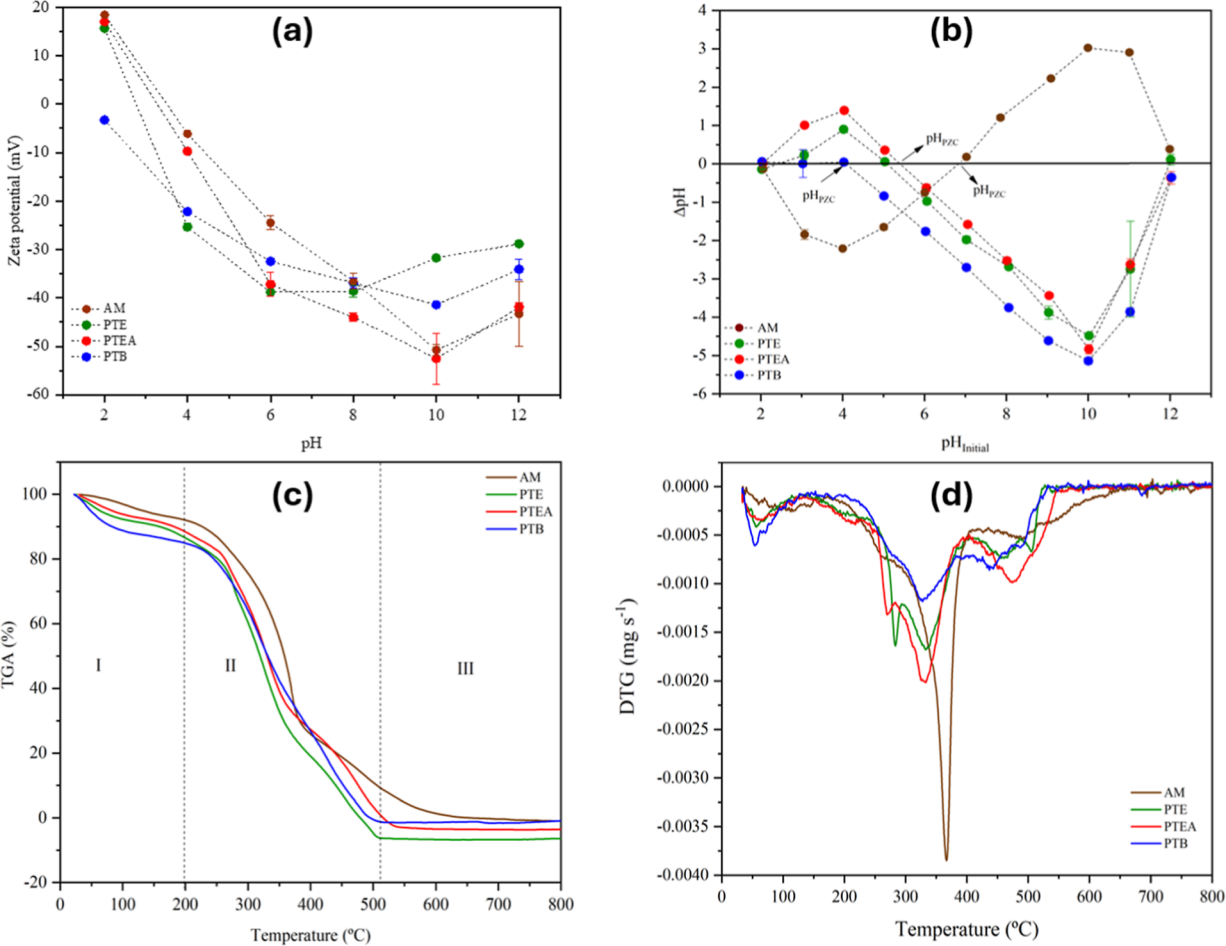
(a) Zeta potential, (b) pH at point of zero charge (pH_PZC_), (c) thermogravimetric analysis (TGA), and (d) derivative thermogravimetry (DTG) curves of pequi almond (AM), thorny endocarp (PTE), thorny endocarp with almonds (PTEA), and pequi tree bark (PTB).

According to Kang et al.,^46^ colloidal dispersions with absolute ZP values above 30 mV are stable, whereas lower values indicate instability and aggregation tendency. In this work, ZP values near or above ±30 mV at pH > 4 suggest stable colloidal dispersions, likely due to the materials’ structure, molecular weight, and predominantly negative charge. These findings indicate enhanced efficiency of the materials in adsorption and coagulation under acidic conditions. Therefore, they have potential applications in removing pollutants such as heavy metals, dyes, pharmaceuticals, and phosphates from wastewater via adsorption and coagulation mechanisms.

The point of zero charge (pH_PZC_), where the material’s surface charge is neutral, was determined from the intersection of ΔpH and initial pH (Figure 4b). The pH_PZC_ values were 5.06 for PTE, 5.38 for PTEA, 4.03 for PTB, and 6.83 for AM. Below the pH_PZC_, surfaces are positively charged and favor anion adsorption; above it, surfaces are negatively charged, promoting cation adsorption. This trend aligns with Zeta Potential data (Figure 4a), showing positive values at pH 2 (15.67 mV for PTE, 17.01 mV for PTEA) and negative values at pH 12 (−28.81 mV and −41.91 mV, respectively). PTB remained negatively charged across the pH range, becoming negative above pH 4.03 due to dissociation of surface groups like carboxylic and phenolic moieties.^28^ In contrast, AM presented a higher pH_PZC_ (6.83), indicating a relatively more basic surface, likely related to its higher content of proteins and lipids. This suggests that AM tends to remain neutral or positively charged up to near-neutral pH, thus favoring anion adsorption over a broader range. These pH_PZC_ values are comparable to those reported for other agro-industrial residues such as babassu mesocarp (4.0), dragon fruit peel (4.3), and açaí pit (5.09), indicating similar surface charge behavior.^47,48^

### 3.5 Thermogravimetric Profile of *C. brasiliense* Byproducts (TGA/DTG)

The TGA/DTG curves of *C. brasiliense* byproducts (Figure 4c,d) reveal distinct thermal behaviors among the lignocellulosic materials, with no significant differences in overall profiles. Stage I (25–200 °C) showed initial mass loss (∼12% for PTB, <10% for PTE, PTEA, and AM), due to moisture evaporation and volatile release.^49^ Stage II (200–550 °C) featured major decomposition of hemicellulose (190–300 °C, peak ∼ 260 °C for PTE/PTEA), cellulose (250–350 °C, peak ∼ 340 °C), and lignin.^50^ AM showed a prominent peak at 380 °C with a higher decomposition rate, attributed to its unique composition and high volatile content,^51^ while PTB’s smaller peak matched its lower volatile content (Table 2). Stage III (>500 °C) corresponded to lignin’s final degradation and related compounds such as flavonoids and phenolics.^52^ Overall, AM exhibited lower thermal stability and higher reactivity, whereas PTE, PTEA, and PTB showed more gradual decomposition, linked to higher lignin and lower volatiles (Tables 1 and 2).

### 3.6 Textural Properties of *C. brasiliense* Byproducts

Nitrogen adsorption–desorption analyses were performed, and Figure 5 displays the isotherm curves for the *C. brasiliense* byproducts. According to IUPAC classification, all materials exhibit combined type II and IV isotherms, indicating the presence of macropores and mesopores. The observed type H3 hysteresis loops at P/P_0_ > 0.5 suggest nonrigid aggregates with plate-like particles and slit-shaped pores.^53^ Table 3 presents the textural properties, revealing very low specific surface areas (0.316 to 0.712 m^2^ g^–1^), likely due to the compact structure of hemicellulose, cellulose, and lignin. The isotherm profiles, combined with low surface area and pore volume, indicate limited macroporosity.^54^ Thus, these materials are low-porosity, and enhancing pore development and surface area would require chemical or physical activation to increase microporosity.^32^

**Figure 5:**
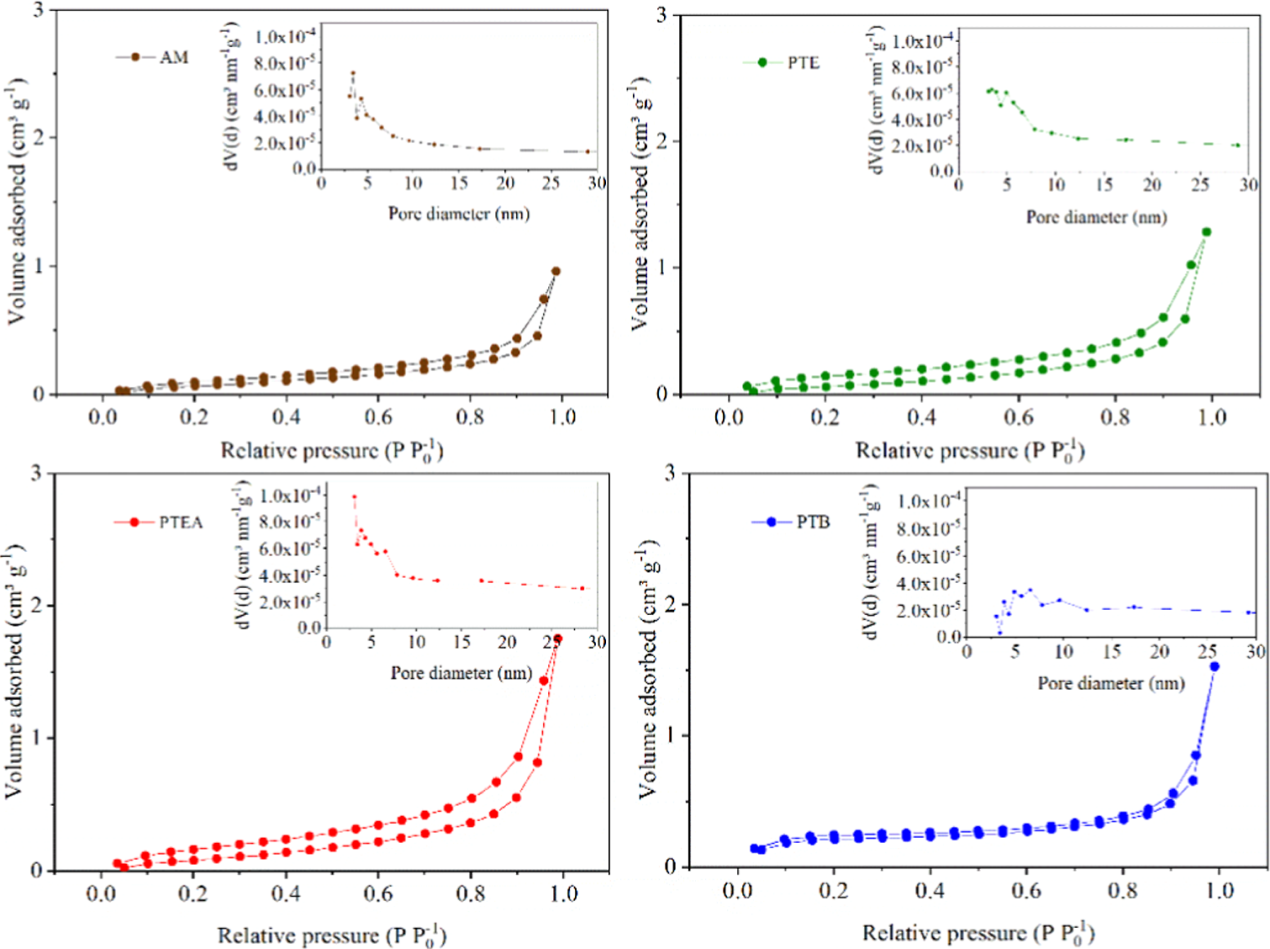
N_2_ adsorption isotherms and pore diameter distribution of pequi almonds (AM), pequi thorny endocarp (PTE), pequi thorny endocarp + almonds (PTEA) and pequi tree bark (PTB).

**Table 3:** Textural Characteristics of Pequi Almonds (AM), Pequi Thorny Endocarp (PTE), Pequi Thorny Endocarp + Almonds (PTEA) and Pequi Tree Bark (PTB)

sample	surface area (m^2^ g^–1^)	pore diameter (nm)	pore volume (cm^3^ g^–1^)	pore width (nm)
AM	0.34	3.42	0.0015	4.89
PTE	0.32	3.42	0.0019	4.89
PTEA	0.44	3.06	0.0027	5.48
PTB	0.71	6.56	0.0021	2.34

The pore diameter distributions (inset of Figure 5) ranged from 2.34 to 5.48 nm, with average pore diameters between 3 and 6.56 nm, confirming the predominance of mesopores. AM and PTE exhibited pores mainly between 3 and 5 nm, while PTB and PTEA also showed mesoporous structures in the 5–10 nm range.

This distribution, along with the presence of macropores, favors the adsorption of pollutants and the retention of suspended particles. Although coagulation primarily involves mechanisms such as charge neutralization and particle bridging, porous morphology may act as a complementary factor, facilitating physical and electrostatic interactions with impurities.^55^

### 3.7 Total Phenolic Content (TPC)

Figure 6 shows the results for the TPC contents in the extracts of AM, PTE, PTEA, and PTB. Among the byproducts analyzed, PTB exhibited the highest TPC levels (157.13 mg_GAE_ g^–1^), indicating a substantial concentration of bioactive compounds. This value is comparable to that reported Bin Mokaizh et al.,^56^ for the bark extract of *C. gileadensis* (166.41 mg_GAE_ g^–1^), obtained at 4 min using 60% ethanol. In contrast, Tomasi et al.^22^ recorded a huge higher value of 354 mg_GAE_ g^–1^ in *Eucalyptus globulus* bark extract, under optimized extraction conditions (141 °C, 15 s). These comparisons emphasize that both biomass composition and extraction parameters strongly influence the phenolic yield, which is particularly relevant for PTB, as it showed the highest performance among the byproducts evaluated.

**Figure 6:**
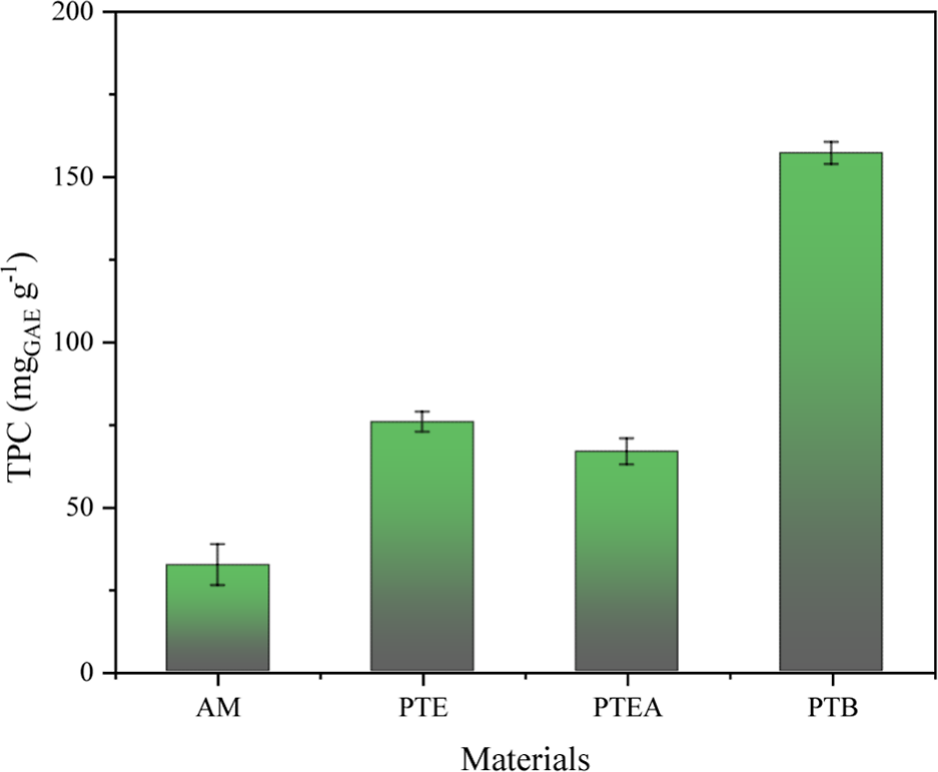
Extraction of total phenolics compounds from the materials. Conditions: 30 mL g^–1^; 105 °C, 50% ethanol proportion and 4 min.

These results can be explained by the chemical and structural composition of the biomass, particularly the higher content of holocellulose, lignin, and structural carbohydrates (glucan, xylan, and mannan) in PTB compared to the other materials. Stanek-Wandzel et al.^57^ demonstrated that the use of specific enzymes such as cellulase, pectinase, and hemicellulase can significantly enhance the extraction yield of phenolic compounds from grape pomace, highlighting the relevance of biomass composition and extraction conditions in process efficiency.

The greater lignocellulosic content and lower proportion of interfering substances in PTB promote the release of phenolic compounds. Moreover, the interaction between phenolics and proteins significantly affects extraction efficiency. As noted by Jobstl et al.,^58^ phenolic–protein interactions can reduce phenolic solubility through complex formation. In this work, PTB, with the lowest protein content, achieved the highest phenolic yield, whereas AM, with the highest protein content, showed the lowest, suggesting that proteins may compete with phenolics during extraction. Noncovalent forces such as hydrophobic interactions, hydrogen bonding, and van der Waals forces can mediate this competition. Additionally, the type of solvent plays a key role, as highlighted by Silva et al.^21^

Lipids also influence phenolic extraction, mainly through hydrophobic interactions. According to Jakobek,^59^ polyphenols may associate with lipids, forming complexes that reduce their solubility in aqueous solvents. In this work, AM had the highest lipid content (47.82%) and the lowest TPC yields, likely due to such interactions. Conversely, PTB, with only 2.03% lipids, exhibited the highest yields, suggesting that a lower lipid content facilitates phenolic availability and extraction by reducing the affinity of phenolics for a lipophilic matrix, thereby enhancing their solubilization and recovery.

The high concentration of phenolic compounds in PTB may play a significant role in contaminant removal, primarily due to their interactions with charged species in solution. Tannins, as effective proton donors, exhibit an anionic nature resulting from the deprotonation of abundant phenolic groups and electron delocalization within the aromatic ring. As suggested by Jeon et al.^60^ the hydroxyphenyl groups in polyphenols can interact with both positively and negatively charged ions through dipolar forces generated by the high electronegativity of oxygen and the release of protons in aqueous media. This behavior is essential for processes such as adsorption and coagulation. Therefore, the high phenolic content in PTB indicates strong potential for application in such systems.

### 3.8 FTIR and SEM of the Extracted Materials (TCP)

FTIR spectra (Figure 7) were analyzed to identify changes in functional groups associated with phenolic compounds in the biomass and in the microwave-assisted extracts of the PTE, PTEA, and PTB materials. The AM sample was excluded from the analysis due to its low phenolic content.

**Figure 7:**
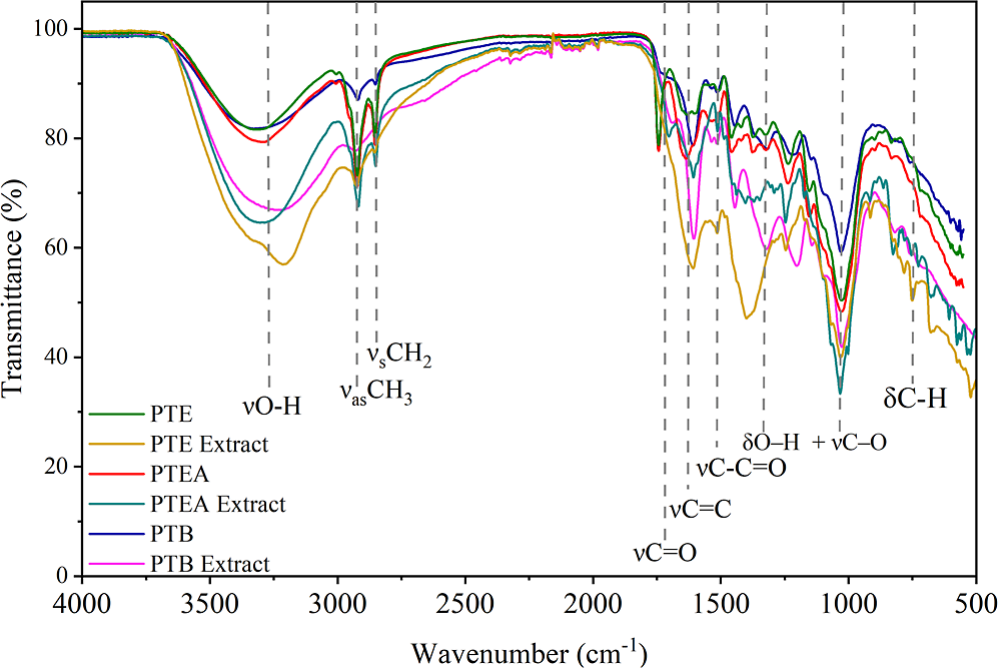
FTIR spectra of pequi tree bark (PTB), pequi thorny endocarp (PTE), and pequi thorny endocarp with almonds (PTEA) before and after microwave-assisted extraction.

A broad band was observed around ∼3300 cm^–1^, attributed to the stretching vibration of hydroxyl (−OH) groups, characteristic of alcohols, phenols, and tannins. This band was more intense in the PTB extract, indicating a higher concentration of hydroxylated bioactive substances such as gallic and tannic acids Ben-Ali et al.^61^ in agreement with the extraction results shown in Figure 6. A decrease in the band between 3200 and 3350 cm^–1^, typically related to hydroxyl groups in gallic acid, was also noted. Bands corresponding to N–H and O–H stretching—typical of phenolic acids such as tannic, gallic, and ellagic acids—were also detected.^62^

Small bands in the 2950–2850 cm^–1^ region were assigned to C–H stretching vibrations of aliphatic groups (methyl, methylene, and methoxy), found in carboxylic acids and polyphenolic compounds.^7^ A small peak between ∼1700 and 1730 cm^–1^, observed only in the PTB sample, was attributed to the stretching of carbonyl groups (C=O). The ∼1610 cm^–1^ band corresponds to the stretching of aromatic rings (νC=C).^63^

Bands between 1500 and 1450 cm^–1^ were related to C–C=O stretching vibrations, while the region from 1300 to 1100 cm^–1^ corresponded to O–H bending, typical of compounds such as gallic acid, quercetin, rutin, and tannic acid. These bands were more intense in the PTB extracts, indicating a higher residual content of such compounds.^63^ The strong band between 1090 and 950 cm^–1^ was attributed to C–O stretching vibrations from carboxylic acids and alcoholic groups (C–OH). The signal between 800 and 750 cm^–1^ was associated with meta-substitution of aromatic protons, indicating the presence of substituted aromatic rings.^62^

The high concentration of phenolic compounds in PTB may play a significant role in contaminant removal, mainly due to their interaction with charged species in solution. Tannins, acting as excellent proton (H^+^) donors, exhibit anionic character as a result of phenolic group deprotonation and electron delocalization across the aromatic ring.^22^

SEM analysis was performed on the solid residues of AM, PTE, PTEA, and PTB after microwave-assisted extraction (MAE) to assess morphological changes induced by the process. These residues, corresponding to the biomass remaining after separation of the liquid extract, are shown in Figure 6. Prior to MAE irradiation (Figure 3), the materials exhibited high structural integrity, with compact, homogeneous surfaces, no visible cracks, and well-organized cell walls. Notably, the PTE sample displayed smooth surfaces and firmly preserved thorns, indicating a mechanically intact plant matrix. Following microwave treatment, substantial morphological changes were observed, likely due to localized thermal stress generated by irradiation. In the PTE sample, for instance, Figure 8a,b reveals severely damaged thorns, with visible cracks, structural discontinuities, and partial collapse of cell walls. Similar but less intense damage was noted in the PTEA sample, while PTB exhibited pronounced cracks, deformations, and collapsed regions, indicative of more extensive cellular rupture. These deformations are attributed to the formation of localized hotspots and steep thermal gradients, characteristic of microwave interaction with polar molecules such as water. As reported by More and Arya,^35^ this phenomenon results from molecular rotation and rapid internal heating, which compromise cellular integrity.

**Figure 8:**
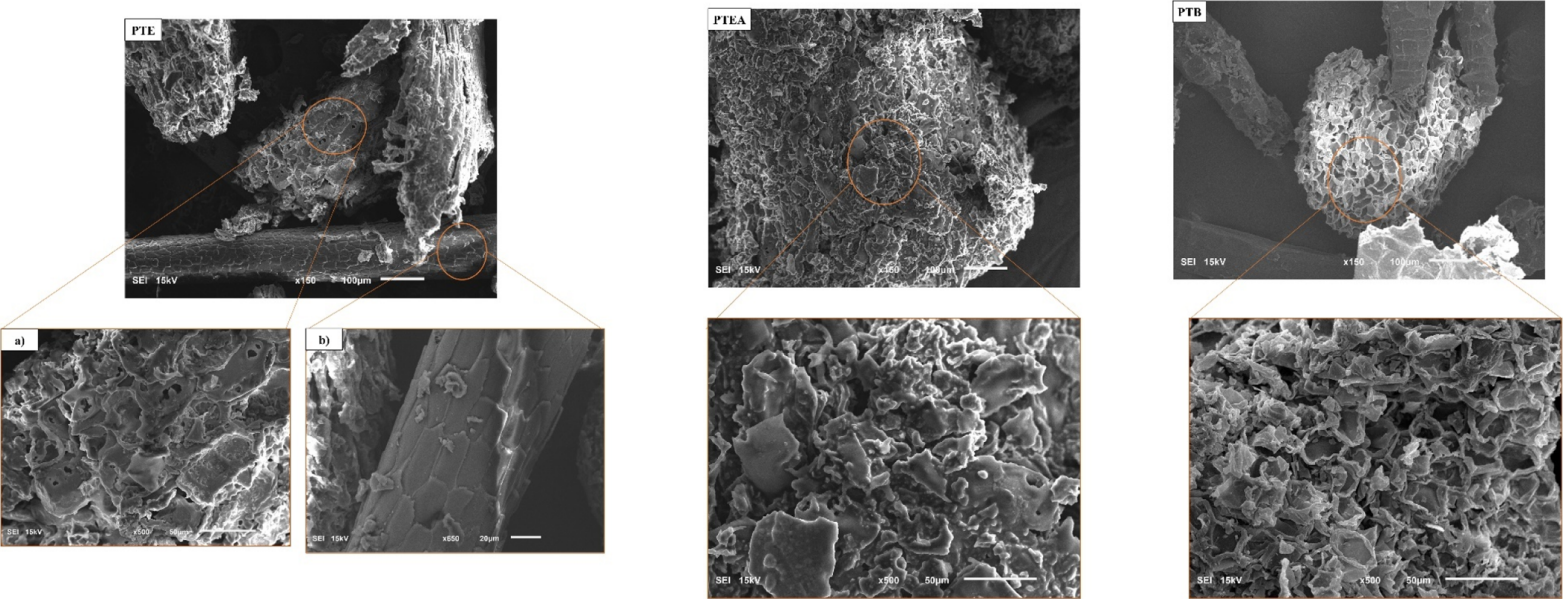
SEM images of pequi tree bark (PTB), pequi thorny endocarp (PTE), and pequi thorny endocarp with almonds (PTEA) samples after microwave-assisted extraction. SEM images of pequi tree bark (PTB), pequi thorny endocarp (PTE), and pequi thorny endocarp with almonds (PTEA) after microwave-assisted extraction. For PTB: (a) general view of the material at ×500 magnification, and (b) detail of the thorn structure at ×650 magnification.

The structural disorganization observed in the micrographs enhances solvent penetration into the plant matrix, thereby improving the extraction of bioactive compounds like tannins and phenolics. Among all samples, PTB exhibited the most significant morphological alterations, which is consistent with its superior extraction efficiency (Figure 6) and FTIR results (Figure 7), confirming higher recovery of phenolic compounds.

Post-MAE SEM images revealed highly rough surfaces, fragmented structures, cavities, and active sites, particularly evident in PTB. This morphology indicates increased porosity, larger surface area, and greater exposure of functional sites—key attributes for adsorption processes. The combination of porous structure, diverse functional groups, and abundant active sites can significantly enhance the adsorptive capacity of these materials, supporting their application in removing dyes, metal ions, and other contaminants from aqueous media.^64^ Thus, the valorization of postextraction residues not only promotes efficient biomass utilization but also aligns with circular economy principles by enabling multiple sustainable applications from a single lignocellulosic feedstock.

## 4 Conclusion

This study revealed distinct physicochemical, thermal, and morphological profiles among the byproducts of the pequi (*C. brasiliense*), including the almond (AM), thorny endocarp (PTE), thorny endocarp with almond (PTEA), and bark of the pequi tree (PTB), highlighting specific and complementary applications in environmental and energy contexts, aligned with the principles of bioeconomy and circular economy.

The bark of the pequi tree (PTB) presented the most favorable profile for environmental applications, standing out due to its high content of phenolic compounds (193.73 mg_GAE_ g^–1^), low lipid concentration, and high proportion of lignin and holocellulose. Its fragmented structure, with a high density of cavities and predominantly mesoporous pores, along with high particle dispersion in a neutral medium, confers a strong affinity for anionic pollutants such as heavy metals, dyes, pharmaceuticals, and phosphates, enabling its use as a natural coagulant and adsorbent.

The materials PTE and PTEA exhibited a lignocellulosic composition similar to that of PTB. Additionally, they exhibited a mesoporous structure and high thermal stability, which can be attributed to their higher lignin content and lower volatile matter. Although they contained lower levels of tannins, these characteristics suggest that both materials can be explored as plant-based coagulants and adsorbents, especially after physicochemical modifications to enhance their specific surface area.

In contrast, the almond (AM) displayed a profile more suitable for energy valorization, with a high lipid content (47.82%), a greater fraction of volatile compounds, and a high calorific value (27.54 MJ kg^–1^), making it a promising feedstock for bio-oil and biofuel production via pyrolysis or transesterification routes.

Finally, it is noteworthy that these byproducts, even after the extraction process, retained a rough morphology and exposed active sites, indicating their potential reuse as adsorbents. This cascading use strategy—bioactive compound extraction followed by environmental application—is aligned with circular economy principles, reinforcing the concept of fully valorizing lignocellulosic residues and establishing a technically feasible and sustainable pathway.
